# Lipid Profile of Patients with Acute Myocardial Infarction (AMI)

**DOI:** 10.7759/cureus.4265

**Published:** 2019-03-18

**Authors:** Naresh Kumar, Suresh Kumar, Anil Kumar, Tariq Shakoor, Amber Rizwan

**Affiliations:** 1 Cardiology, Shalamar Hospital, Lahore, PAK; 2 Internal Medicine, Bolan Medical College, Quetta, PAK; 3 Cardiology, Punjab Institute of Cardiology, Lahore, PAK; 4 Family Medicine, Civil Hospital, Karachi, PAK

**Keywords:** serum triglyceride, ldl, lipid profile, acute myocardial infarction, serum cholesterol, hdl

## Abstract

Introduction

Irrespective of underlying hyperlipidemia, the serum lipid profile witnesses a phasic fluctuation immediately after a major cardiovascular event. This study aims to evaluate the change in serum lipid profile in patients with acute myocardial infarction (AMI).

Methods

It was a prospective, cross-sectional study conducted in the department of cardiology, Shalamar Hospital, and Punjab Institute of Cardiology, from October until December 2018, focusing on patients admitted with ST-elevation myocardial infarction (STEMI). The patient's demographics and lipid profile (in mg/dl) within the first 24 hours and after 48 hours of the event were recorded.

Results

The mean serum total cholesterol (TC) levels decreased from 207.5 ± 30.5 to 192.4 ± 49.3 after 48 hours (p-value <0.0001). Mean serum triglyceride (TGs) levels increased from 153.8 ± 10.2 to 183.8 ± 14.8 (p-value <0.0001). Mean serum low density lipid-cholesterol (LDL-C) decreased from 149.0 ± 41.2 to 133.4 ± 54.0 (p-value = 0.0003). Mean serum high density lipid-cholesterol (HDL-C) decreased from 46.6 ± 9.9 to 40.7 ± 11.8 (p-value <0.0001).

Conclusion

Phasic fluctuations in serum lipid profile are observed after acute myocardial infarction (AMI). The trend that follows include reduced TC, LDL-C, and HDL-C, and increased TGs. Periodic lipid profile must be evaluated in all patients admitted for AMI to understand the changing trend, initiate lifestyle measures to reach target lipid levels, and predict the choice of lipid-lowering therapy.

## Introduction

Acute myocardial infarction (AMI) is by far the most important form of ischemic heart disease (IHD), and it alone is the leading cause of death in the United States (US). About 1.5 million individuals in the US suffer from acute MI annually and approximately one-third of them die [1-2].

The severity of IHD and its resultant mortality can be reduced by controlling modifiable risk factors. Among the modifiable risk factors of IHD--hypertension (HTN), diabetes mellitus (DM), cigarette smoking, dyslipidemia, and severe obesity--dyslipidemia (hyperlipidemia and hypercholesterolemia) has been given the greatest attention recently [3]. Epidemiological surveys have shown that atherosclerosis due to dyslipidemia is directly correlated with a risk of IHD. Coronary artery disease (CAD) has been directly linked to hypercholesterolemia, particularly elevated plasma levels of cholesterol in low-density lipoproteins (LDL-C) [4-5]. Increased risk of AMI has been seen in patients with low plasma levels of high-density lipoprotein (HDL-C) cholesterol [6].

In the Pakistani population, a recently published study reported that 63% of the population had one or more deranged lipid fraction. The common, isolated forms of lipid irregularity were low HDL-C (17.3%) and high triglyceride (TG) (11.2%) [7]. Both of these parameters have been regarded as linear risk factors for CAD and stroke [8-9]. In a study with young patients of AMI (within 24 hours), it was seen that 60.83% were dyslipidemic; the most common isolated deranged lipid fraction was TG (45%) whereas low HDL was least common (10.83%) [10].

This study aims to evaluate the change in serum lipid profile in patients with AMI which will impact the choice of lipid-lowering therapy in these patients.

## Materials and methods

It was a prospective, cross-sectional study conducted simultaneously in the department of cardiology, Shalamar Hospital, and the Punjab Institute of Cardiology, from October till December 2018. All the patients admitted with ST-elevation MI (STEMI) were included after receiving informed consent. STEMI was diagnosed by pertinent history, electrocardiogram (ECG), and cardiac biomarkers. The excluded patients included those already taking lipid-lowering drugs, who presented after 24 hours of MI, and who had previously been diagnosed with hyperthyroidism. The reason for excluding patients with these comorbidities was to reduce the bias in the results. 

Along with all other forms of biochemical testing, their lipid profile (in mg/dl) was also routinely evaluated as a hospital protocol. This study did not make any additional interventions and did not cause any extra burden on the patient or hospital resources. The patient’s age, gender, smoking history, co-morbidities including diabetes mellitus and hypertension, previous history of major cardiovascular events (MACE including MI and stroke), and body weight and height were noted for all patients. The body mass index (BMI) was calculated and BMI >30 kg/m2 were termed as 'obese'. Lipid profile within the first 24 hours of the event and after 48 hours was recorded. There were five patients who died before 48 hours of admission and two were shifted to another hospital. All seven patients were replaced with new patients.

Statistical analysis was done using SPSS v. 22.0 (IBM Corporation, Armonk, New York, United States). Continuous variables including age and lipid profile were analyzed via descriptive statistics and were presented as mean and standard deviation (SD) while categorical variables including gender, cardiovascular history, smoking history, and the type of lipid abnormality were presented by percentages and frequencies. Confounding variables including age, gender, cardiovascular history, and smoking history were controlled by stratification chi-square and were utilized for a comparison of frequencies. Means of serum biochemical levels at two-time intervals were compared using paired sample T-test. P-value ≤0.05 was taken as significant.

## Results

There were more men than women (75.6% vs. 24.4%). The mean age of the patients was 53.8 ± 10.2 years. The details of patient characteristics including their ages, co-morbidities, the presence of obesity, and previous history of MACE are shown in Table 1. 

**Table 1:** Demographic characteristics of the patients (n=250) BMI: Body mass index, MACE: Major cardiovascular event (myocardial infarction, stroke)

Patient Characteristics	Frequency (%)
Gender	
Male	189 (75.6%)
Female	61 (24.4%)
Age in years	
25-40	28 (11.2%)
41-50	79 (31.6%)
51-60	82 (32.8%)
61-70	61 (24.4%)
Mean ± SD	53.8±10.2
Co-morbidities	
Hypertension	67 (26.8%)
Diabetes mellitus	59 (23.6%)
Diabetes + hypertension	84 (33.6%)
Obesity (BMI >30 kg/m^2^)	113 (45.2%)
Previous history of MACE	38 (15.2%)

The biochemical lipid profile of the patients within the first 24 hours of the myocardial infarction was noted. On an isolated level, the most commonly deranged parameter was serum TGs - 70% had elevated TGs, followed by 32% patients who had elevated TC, 31.2% who had decreased HDL-C, and 28% who had elevated LDL-C. Of the total, 18% of the patients had both elevated TGs and decreased HDL-C. The mean values and frequencies of elevated and non-elevated lipid profile parameters are shown in Table 2. After 48 hours, the biochemical profile of the patients was repeated. A slight dip was seen in TCs, LDL-C, and HDL-C; however, TGs increased (Table 2).

**Table 2:** Biochemical lipid profile (mg/dl) of the patients within the first 24 hours of STEMI (n=250) LDL-C: Low-density lipid-cholesterol, HDL-C: High-density lipid-cholesterol, TGs:  Triglycerides, STEMI: ST-elevation myocardial infarction

Parameters of Lipid Profile	Frequency (%) (n=250)			Mean ± SD		
	Within 24 h	After 48 h		Within 24 h		After 48 h
Serum total cholesterol (mg/dl)						
Elevated	80 (32%)	89 (35.6%)			207.5 ± 30.5	192.4 ± 49.3
Non-elevated	170 (68%)	161 (64.4%)				
Serum triglyceride (mg/dl)						
Elevated	164 (65.6%)	192 (76.8%)			153.8 ± 10.2	183.8 ± 14.8
Non-elevated	86 (34.4%)	58 (23.2%)				
Serum LDL-C (mg/dl)						
Elevated	70 (28%)	83 (33.2%)			149.0 ± 41.2	133.4 ± 54.0
Non-elevated	180 (72%)	167 (66.8%)				
Serum HDL-C (mg/dl)						
Decreased	83 (33.2%)	83 (33.2%)			46.6 ± 9.9	40.7 ± 11.8
Non-decreased	167 (66.8%)	167 (66.8%)				
Combine deranged TGs and HDL (mg/dl)						
Deranged	45 (18%)		69 (27.6%)			
Not-deranged	205 (82%)		181 (72.4%)			

**Figure 1:**
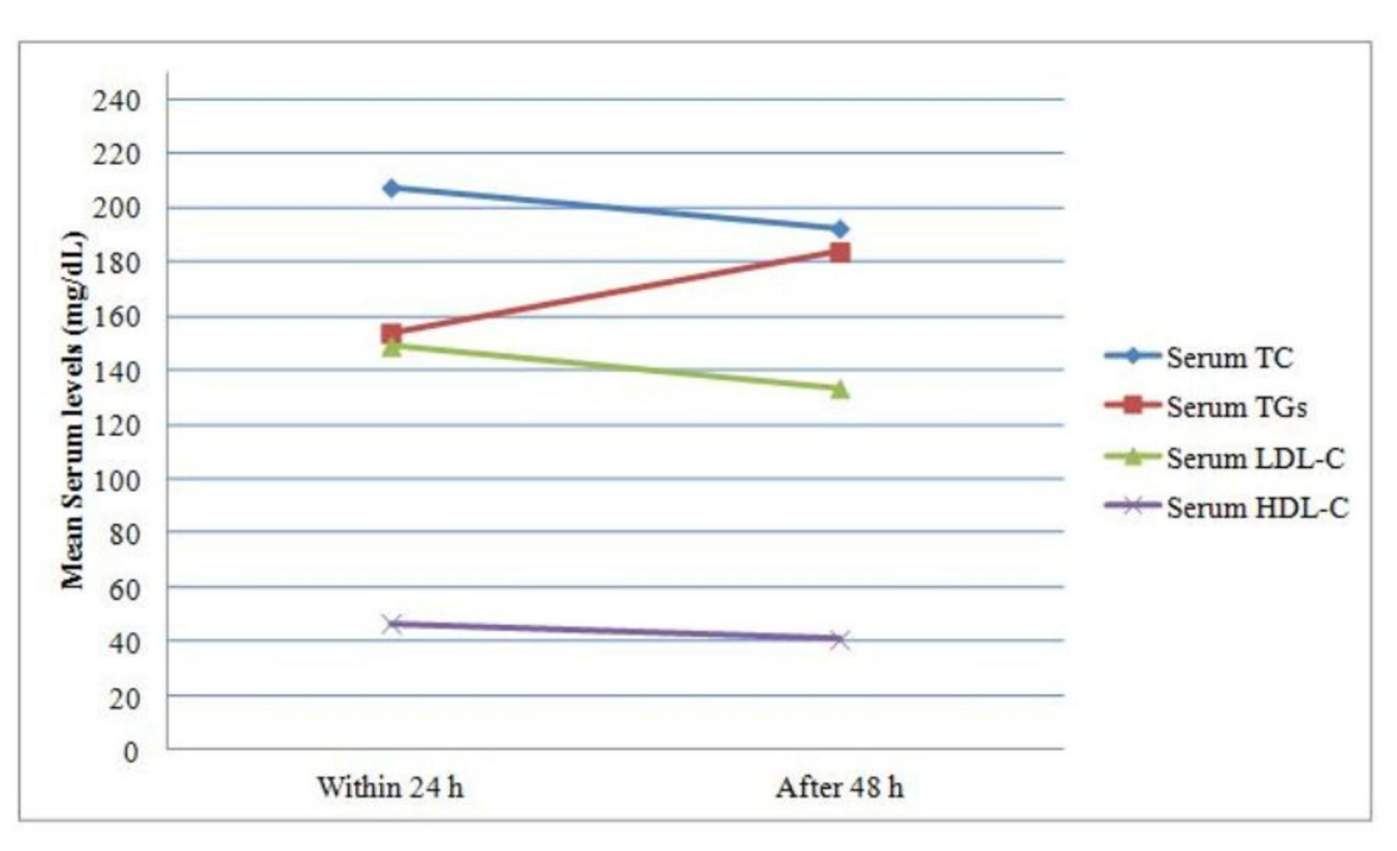
Mean change in the serum lipid profile (mg/dl) from the first 24 h to 48 h (n=250) LDL-C: Low-density lipid-cholesterol, HDL-C: High-density lipid-cholesterol, TGs: Triglycerides, TC: Total cholesterol

The mean change in serum lipid profile over time is shown in Figure 1.

The lipid profiles of the patients were compared at both time instances (within 24 h vs. after 48 h). The frequency of isolated deranged TGs, and deranged combined TGs and HDL-C, was statistically significant after 48 h of acute MI. The mean serum levels of serum TC, serum LDL-C, and HDL-C decreased significantly, whereas serum TGs increased as shown in Table 3.

**Table 3:** Comparison of frequencies and mean of biochemical lipid profile (mg/dl) of the patients within the first 24 h and after 48 h (n=250) LDL-C: Low-density lipid-cholesterol, HDL-C: High-density lipid-cholesterol, TGs:  Triglycerides, TC: Total cholesterol

Biochemical profile (mg/dl)	Within first 24 h (n=250)	After 48 h (n=250)	P value
Comparison of frequencies			
Elevated TC	80 (32%)	89 (35.6%)	0.39
Elevated TGs	164 (65.6%)	192 (76.8%)	0.01
Elevated LDL-C	70 (28%)	83 (33.2%)	0.20
Decreased HDL-C	83 (33.2%)	83 (33.2%)	1.0
Combined deranged TGs and HDL-C	45 (18%)	69 (27.6%)	0.01
Comparison of mean ± SD			
Serum TC	207.5 ± 30.5	192.4 ± 49.3	<0.0001
Serum TGs	153.8 ± 10.2	183.8 ± 14.8	<0.0001
Serum LDL-C	149.0 ± 41.2	133.4 ± 54.0	0.0003
Serum HDL-C	46.6 ± 9.9	40.7 ± 11.8	<0.0001

## Discussion

Major cardiovascular events are the leading cause of death globally. Extensive research has been done over the decades to understand the extent of severity and fatality of the disease in order to control its effects and prevent mortality. Keen interest has been shown in taking measures to minimize the modifiable risk factors for MACE. One of the most crucial relationships of cardiovascular illnesses is with diet and lifestyle habits including the proportion of carbohydrates and fat intake, the extent of physical activity, day-to-day stress, and stress coping mechanisms. In this regard, researchers have shown a keen interest in the relationship of serum lipid profile with cardiovascular disease. This study highlighted that in patients with MACE, the most common deranged parameter of lipid profile was serum triglycerides.

In a LUNAR trial conducted with patients of STEMI, a slight dip in the biochemical lipid profile was seen on the first and second day after STEMI which again rose on the fourth day. Being informed of the fluctuation in the biochemical profile helps in making the right decisions for lipid-lowering therapy [11].

The modification of the lipid profile early after acute myocardial infarction was first reported by Biorck et al., back in 1957 [12]. Since then, although a change in lipid levels has been reported repeatedly, the data is not consistent. In an earlier study, it was reported that serum TC may decrease up to 47%, serum LDL-C up to 39%, serum HDL-C up to 11%, and serum TGs may increase by almost 50% [13]. After that, in another review, it was reported that serum TC may decrease by 1.25%-47%, serum LDL-C by 1.7%-39%, serum HDL-C may fall by 0%-11% and serum TGs may increase by 9.8%-50% [14]. In an Indian study that followed its patients for three months, serum TC and LDL-C did not show any significant change during the hospital stay and even at the three-month follow-up. However, a gradual fall in HDL-C was seen from the second day onwards, with the pre-discharge rate seeing a statistically significant reduction, which remained consistent at a three-month follow-up visit. The LDL-C/HDL-C ratio was significantly increased at pre-discharge as compared to the first day. TGs also significantly increased on the third day of MI [15].

Though almost all researchers and clinicians have agreed with the trend that serum LDL, HDL, and TC rise and serum TGs fall immediately after acute MI, there is still little understanding of the extent of change and the time taken after the event for the changes to reach the maximum and return to baseline. The changes are assumed to first appear within 24-48 hours after MI, reach their peaks in 4-7 days, and then subside after some months. However, the extent and duration of the change in lipid levels are majorly determined by the severity of infarction and tissue necrosis and serum lipid levels before the event [16]. A consensus has also not been achieved as to whether or not therapeutic interventions including thrombolytic treatment and percutaneous coronary interventions influence changing lipid levels [16-17].

The lipid profile must be assessed in every patient admitted with acute coronary syndrome within the first 24 hours and then periodically until a steady healthy state is achieved. The change within the first 24 hours is minimal, and then phasic changes follow. Hence, the first measurement can serve as a relatively reliable source to inform the selection of the lipid-lowering therapy. However, correctly recognizing the baseline is still difficult. Since a decreasing trend in TC, LDL, and HDL is seen periodically after MI, lipid-lowering therapy must be initiated even if the results are within the physiological range in the first few days [18].

## Conclusions

In patients admitted with acute coronary syndrome, serum lipid profile evaluation must be mandatory within the first 24 hours of admission. Phasic fluctuation may be observed for a short period of time, but the trend that follows includes reduced TC, LDL, and HDL, and increased TGs. Periodic evaluation of the lipid profile helps in understanding the changing trend, initiating lifestyle measures to reach target lipid levels, and predicting the choice of lipid-lowering therapy.
